# Role of the radiologist in the diagnosis and management of the two forms of hepatic echinococcosis

**DOI:** 10.1186/s13244-022-01190-y

**Published:** 2022-04-08

**Authors:** Paul Calame, Mathieu Weck, Andreas Busse-Cote, Eleonore Brumpt, Carine Richou, Celia Turco, Alexandre Doussot, Solange Bresson-Hadni, Eric Delabrousse

**Affiliations:** 1 Department of Radiology, University of Bourgogne Franche-Comté, University Hospital Besançon, 3 Boulevard Fleming, 25030 Besançon, France; 2 EA 4662 Nanomedicine Lab, Imagery and Therapeutics, University of Bourgogne Franche-Comté, Besançon, France; 3 Department of Hepatology, University of Bourgogne Franche-Comté, University Hospital Besançon, 25030 Besançon, France; 4 Department of Digestive Surgery, University of Bourgogne Franche-Comté, University Hospital Besançon, 25030 Besançon, France; 5 Laboratoire de Parasitologie-Mycologie, University Hospital Besançon, 25030 Besançon, France; 6 Centre National de Référence Echinococcoses, University Hospital Besançon, 25030 Besançon, France

**Keywords:** Echinococcosis, Radiology (Interventional), Radiologists, Diagnostic imaging, Alveolar echinococcosis, Cystic Echinococcosis

## Abstract

Echinococcosis is a parasitic disease caused by two zoonotic tapeworms (cestodes) of the *Echinocococcus* genus. It can be classified as either alveolar or cystic echinococcosis. Although the two forms differ significantly in terms of imaging findings, they share similarities in terms of management and treatment. In parallel to medical treatment with albendazole (ABZ), and surgery, historically used in these diseases, various imaging-guided interventional procedures have recently emerged (drainage, stenting, or Puncture, aspiration, injection, and reaspiration (PAIR)). These options open up a new range of therapeutic options. As in oncology, multidisciplinary consultation meetings now play a major role in adapted management and patient care in hepatic echinococcosis. Consequently, diagnostic imaging and interventional expertise have brought radiologists to the fore as important members of these multidisciplinary team. The radiologist will need to evaluate parasite activity in both forms of the disease, to guide the choice of the appropriate therapy from among medical treatment, interventional radiology procedures and/or surgical treatment. Knowledge of the specific complications of the two forms of echinococcosis will also help radiologists to discuss the appropriate treatment and management. The aim of this review is to describe the core knowledge that what a radiologist should possess to actively participate in multidisciplinary meetings about hepatic echinococcosis. We discuss the role of imaging, from diagnosis to treatment, in alveolar (AE) and cystic echinococcosis (CE), respectively.

## Key points

- Imaging has a central role in the positive diagnosis of alveolar and cystic echinococcosis.
- Assessment of parasitic activity is crucial in both forms of echinococcosis and requires specific imaging techniques.
- Percutaneous interventional procedures are used in case of complications of alveolar echinococcosis and to treat some forms of cystic echinococcosis.

## Introduction

**Fig. 1:**
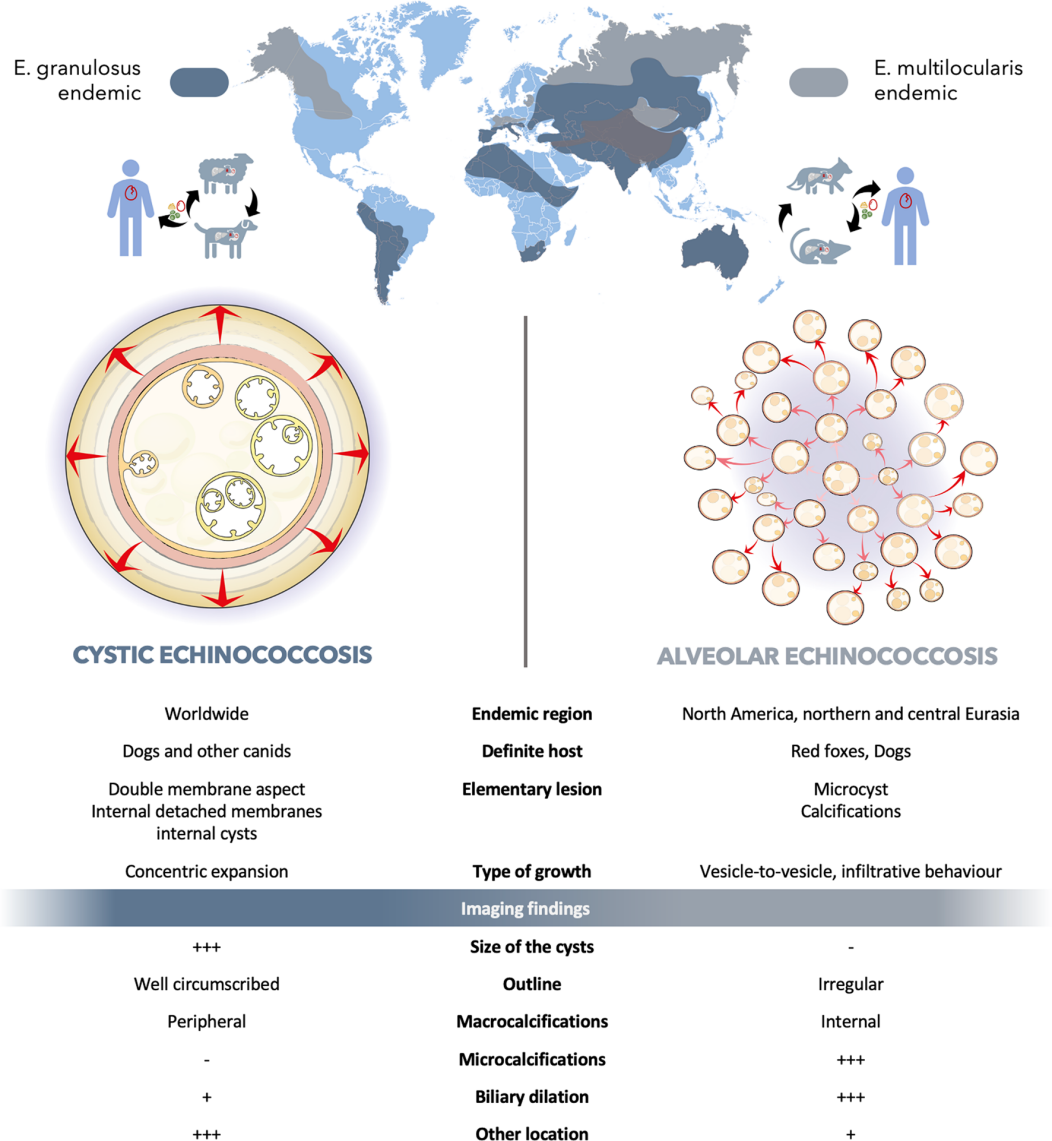
Figure representing type of growth, main demographic and imaging findings of cystic (CE) and alveolar echinococcosis (AE)

**Fig. 2:**
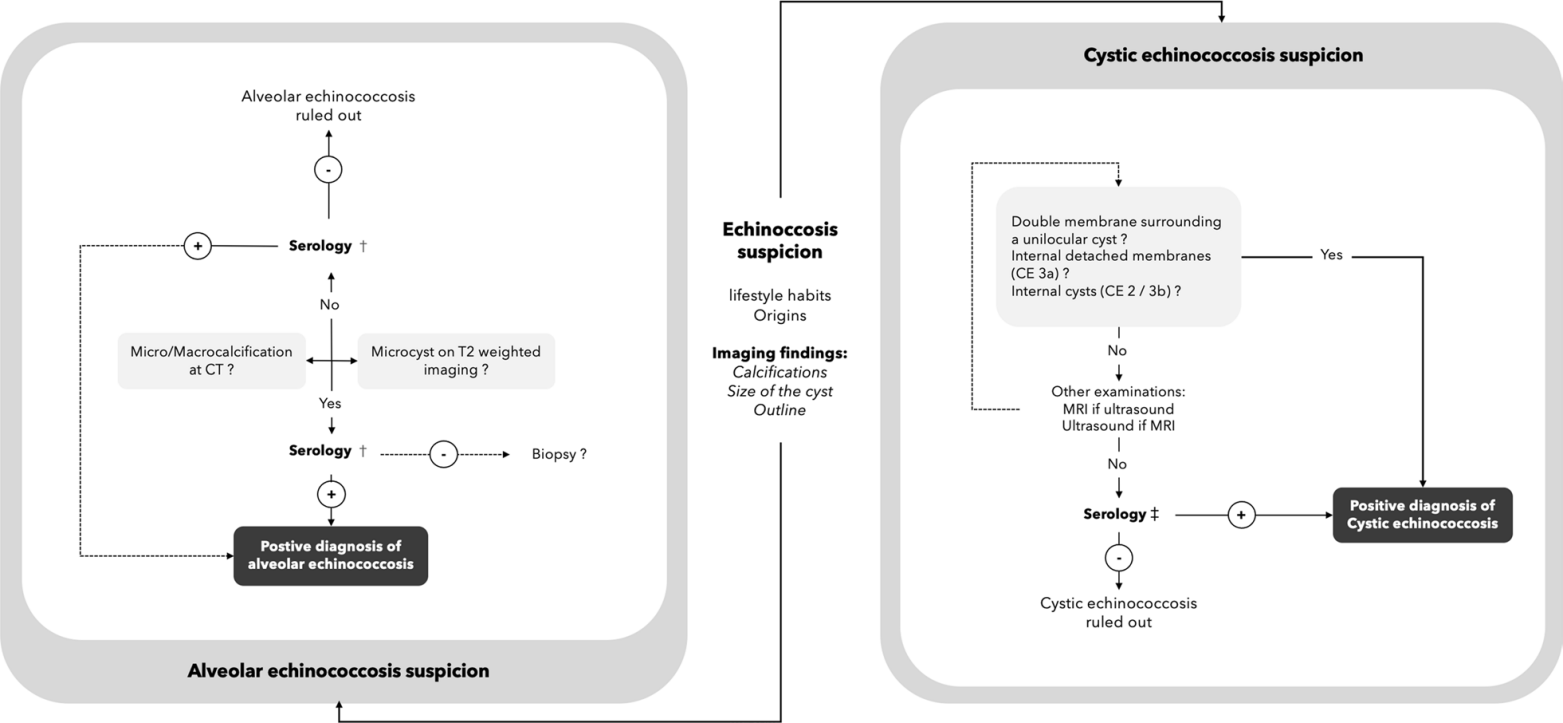
Role of imaging in the positive diagnosis of CE and AE. †First-line tests generally comprise EgHF-ELISA or indirect hemagglutination. The confirmation test in combination with the Em2-and rec-Em18-ELISA enables diagnosis with sensitivity of almost 100%. ‡ Western blotting is the more specific test, used as a confirmatory test in addition to ELISA and IHA (sensitivity varies according to CE stage)

**Fig. 3:**
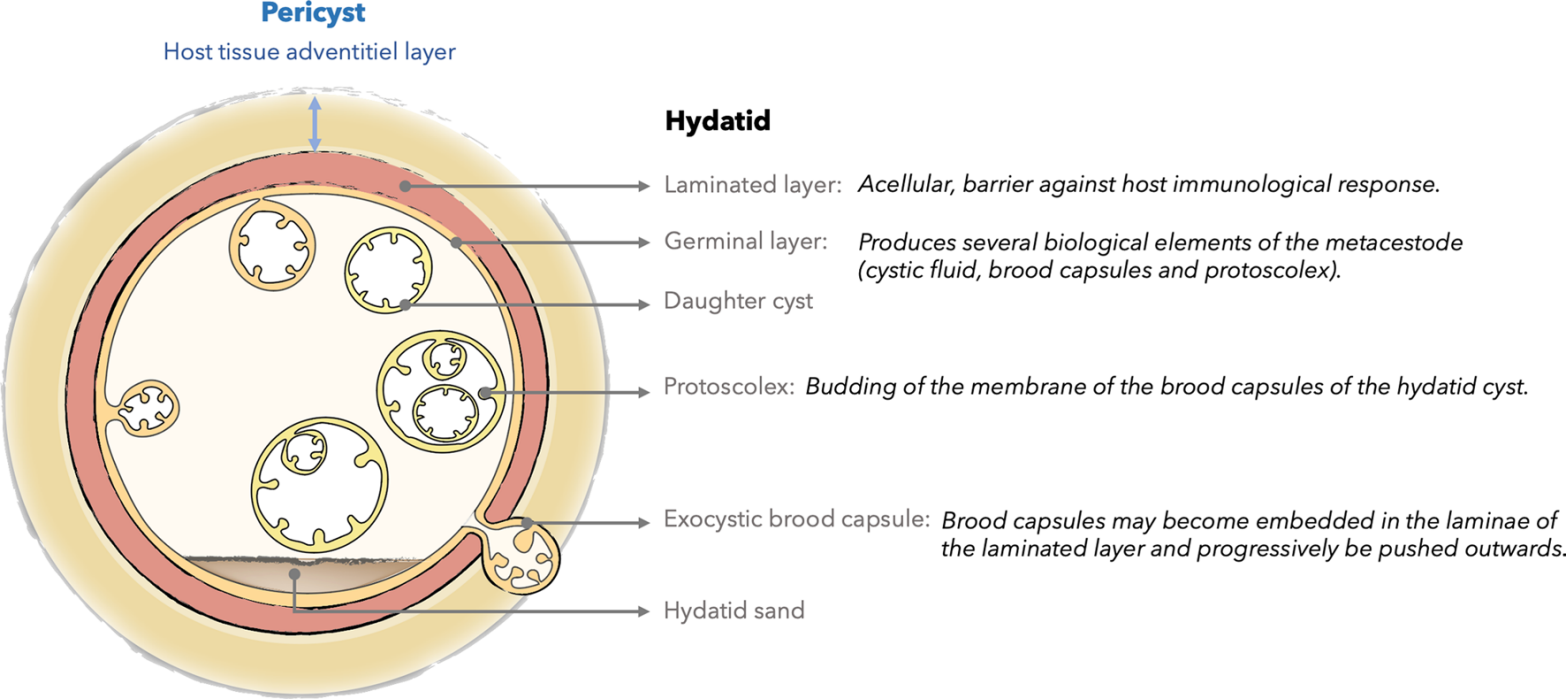
Schematic structure of a cystic echinococcosis cyst

Echinococcosis is a zoonosis caused by cestodes of the genus *Echinococcus* (family Taeniidae). It refers principally to two severe zoonotic tapeworm (cestodes) diseases, namely alveolar echinococcosis, caused by *Echinococcus multilocularis*, and cystic echinococcosis, caused by *Echinococcus granulosus *sensu lato [1]. Although these zoonoses are from the same family, they differ greatly in terms of imaging findings (Fig. 1), but share similarities in terms of management (Fig. 2) and treatment. The differences in terms of imaging findings are principally due to their type of growth. CE is characterized by concentric expansion that leads to a round and cystic lesion, without infiltration of the adjacent parenchyma. CE lesions are surrounded by a stratified layer that contains the parasitic growth inside the germinal membrane (Fig. 3). The germinal membrane of AE is not protected from its host by a stratified layer and is much more fragile. Consequently, it has a vigorous activity that is all the more easily exerted because it is no longer trapped inside the rigid wall of a cyst (unlike the hydatid cyst). These very distinct types of growth—concentric expansion in CE vs vesicle-to-vesicle in AE—explain why these two parasites are so different in terms of imaging findings. Conversely, the two parasites are from the same family, so the medical treatment is the same, and both are liver parasites, with the result that the management strategy may also be similar. This review aims to summarize everything a radiologist should know about these two parasites, which, although different from a radiological point of view, share similarities in terms of management.

## Alveolar echinococcosis

Alveolar echinococcosis (AE) is a rare but invasive disease, caused by infection with the larval stage (metacestode) of the parasite *Echinococcus multilocularis*. The larvae invade various organs but particularly the liver, and infected individuals may remain asymptomatic for years after contamination. The rarity of this infection and its tumor-like pattern of growth and infiltration make the diagnosis challenging (Fig. 1) [2, 3].

In addition to evaluating parasitic activity, imaging plays a significant role in the evaluation of resectability. While magnetic resonance imaging (MRI) and Fluorine-18-Fluorodeoxyglucose Positron Emission Tomography (FDG-PET)/CT scan remain the techniques of choice for the assessment of parasite activity, abdominal CT is the modality of choice for pre-operative evaluation (Table 1) [4].

**Table 1:** Imaging modalities, indications, advantages and disadvantages in AE and CE

	When to use			
	Alveolar echinococcosis	Cystic echinococcosis	Advantages	Disadvantages
US	Diagnosis Incidental diagnosis (Heterogeneous echogenic liver lesion) Follow-up (screening for biliary dilatation or vascular involvement)	Diagnosis Reference for early diagnosis in endemic areas Modality of choice for evaluation of WHO-IWG classification (Internal matrix visualization)	Low cost Availability Radiation free	Operator dependant Limited by body habitus No exhaustive study Lack of extension assessment
CEUS	Diagnosis Confirm absence of enhancement of echogenic liver lesion in case of fortuitous discovery	No indication	Safety	Requires experience
CT	Diagnosis/follow-up Detail number, size and location of AE lesions Preoperative imaging: Assessment of hilar extension to vascular and biliary structures Peripheral contacts (diaphragm, pericardium, peritoneum)	Diagnosis/follow-up Not suitable for staging the disease Modality of choice to detect complications (rupture of CE lesions) Work-up of patients (look for lesions of lung and peritoneum)	Availability Spatial resolution that enables precise preoperative work-up (exploration of the entire peritoneal cavity ± chest	Radiation exposure Requires intravenous contrast injection (usefulness limited in allergic patients, chronic kidney disease)
MRI	Diagnosis/follow-up Modality of choice for positive diagnosis (pathognomonic microcyst) Essential in uncertain cases (non-calcified lesions++) Modality of choice for biliary structure extension assessment Parasitic activity assessment (microcyst in T2 / restricted diffusion in DWI)	Diagnosis/follow-up Modality of choice for biliary complications After US screening: complete locoregional extension assessment Internal matrix characterization if calcified lesion on US	Radiation free Detailed imaging with qualitative and quantitative analysis	Time of examination Availability
FDG-PET	Diagnosis/follow-up Modality of choice to assess disease activity (Peripheral hypermetabolism, Delayed acquisition+++) Monitoring of parasitic activity	No indication	Easily readable	Radiation exposure Availability Limited to the assessment of parasitic activity

### Physiopathology

AE endemic areas are located exclusively in the northern hemisphere, mainly China, North America and Central Europe (southern Germany, Switzerland, western Austria and eastern France). In the life cycle of *Echinococcus multilocularis*, foxes and dogs are the final hosts, while rodents are intermediate hosts. Humans are an accidental intermediate host, contaminated by the ingestion of adult parasite eggs released into the environment by the final host (soil, food, feces, etc.). After ingestion, the eggs hatch and spread oncospheres in the intestine, which pass through the intestinal wall to the portal and lymphatic vessels, and consequently reach the liver. Extrahepatic primary involvement is rare and mainly involves the lung [1, 2, 5, 6].

Secondarily, in the liver, the larval (metacestode) proliferation leads to the growth of a liver mass. Lesions are infiltrative, can be isolated or multiple, and can be voluminous (up to 15–20 cm), with no clear margin between the lesion and healthy parenchyma. Lesions are composed of aggregated vesicles, calcifications, necrotic areas, and are often surrounded by fibrosis and a granulomatous reaction due to the host immune response [6].

The aggregated vesicles (small cysts < 1 cm) give the lesion its multilocular aspect (Fig. 1).

AE develops through progressive peripheral growth of the metacestode in the “germinal layer” (active part of the lesion) within the granulomatous structure, composed of macrophages, lymphocytes, fibroblasts, and neo-vessels. The natural course of the disease consists of approximately 5–15 years of asymptomatic incubation followed by a chronic period [1, 2].

### Imaging findings

The rarity of this infection makes it difficult to diagnose. The diagnosis depends on accurate history-taking, imaging, serology, and sometimes, histopathological analysis. The diagnosis of AE, usually made by imaging, can be fortuitous, or revealed by complications such as abdominal pain or jaundice [2, 6]. Imaging findings of AE are mainly the consequence of the presence of microcystic lesions associated with an infiltrative behavior that may lead to local complications, such as extra-hepatic involvement and biliary dilation.

#### Ultrasonography (US) and contrast-enhanced ultrasonography (CEUS)

**Fig. 4:**
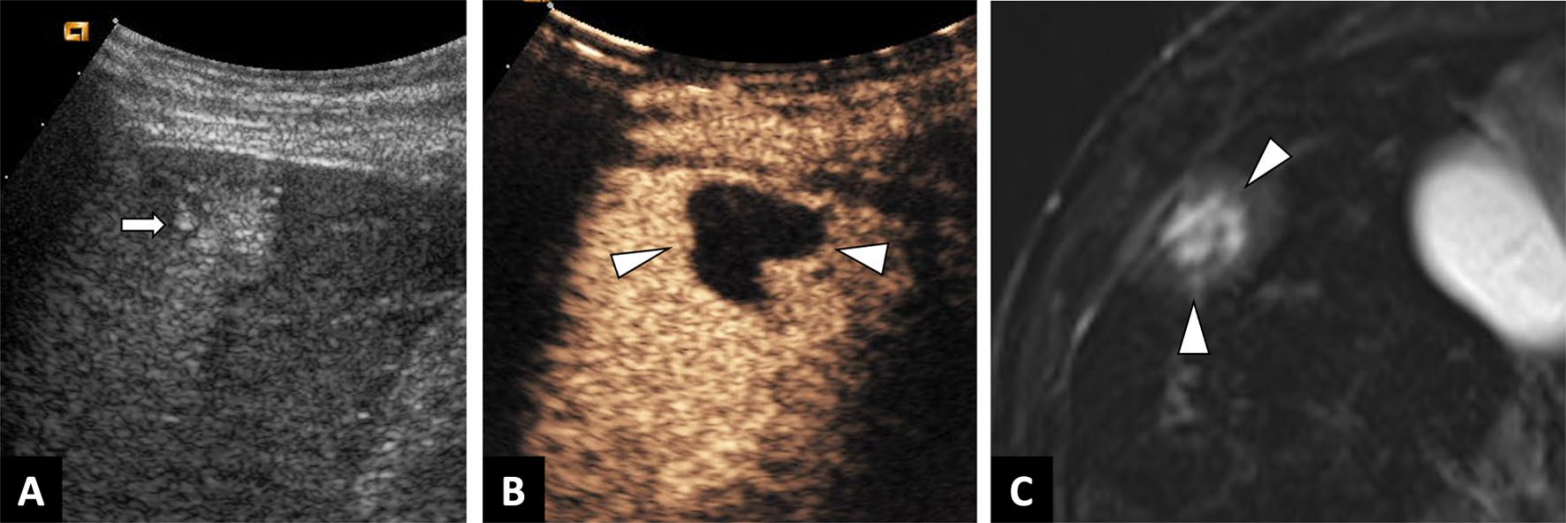
Incidental alveolar echinococcosis discovered in a 65-year-old man with a recent history of acute myeloid leukemia, and presenting with liver blood test abnormalities. **A.** Abdominal ultrasound showing heterogeneous echogenic mass with irregular borders. **B.** Contrast-enhanced ultrasound showing the absence of enhancement of the liver lesion (arrowheads). **C.** T2-weighted MR images in the axial plane showing pathognomonic microcysts (arrowheads)

The typical finding on B-mode ultrasound is a heterogeneous echogenic mass with irregular borders, composed of cystic vesicles due to necrotic areas, calcifications, and surrounded by a ring-like zone of hyperechogenicity that corresponds to fibrosis (Fig. 4).

Less common findings include a mass with multiple hyperechogenic solid lesions, described as a “hailstorm pattern,” which can be confused with hemangioma of the liver, which is much more common [7] and also characterized by acoustic enhancement due to its liquid component. CEUS may be a useful imaging modality when a heterogenous hyperechogenic liver mass is discovered, and will help in differentiating AE from other potential diagnoses. AE will be characterized by an absence of enhancement, or limited peripheral enhancement (Fig. 4) [8].

#### Computed tomography (CT)

**Fig. 5:**
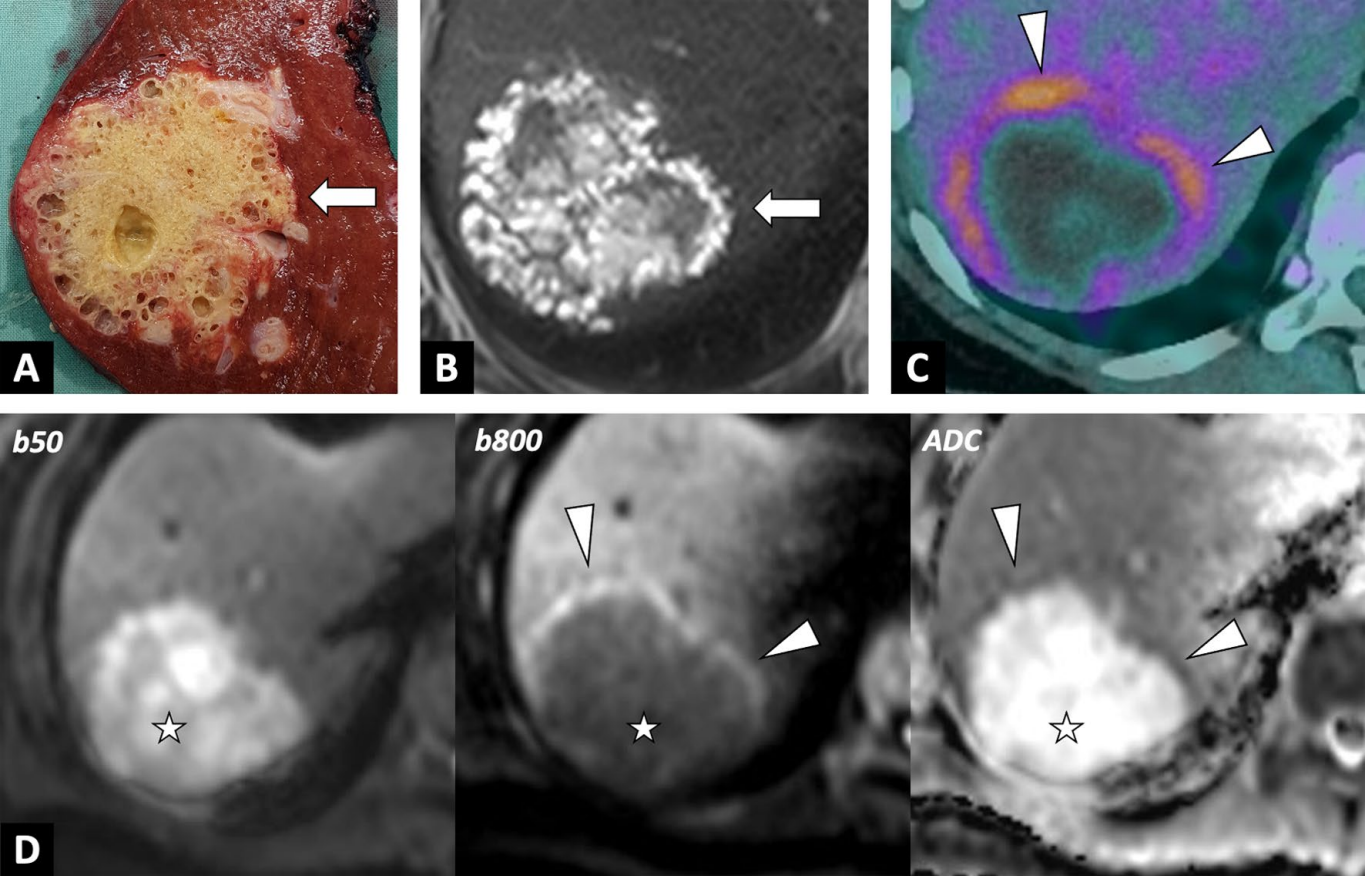
Overall correlations of right liver alveolar echinococcosis in a 54-year-old women. **A** Photograph of gross specimen after right hepatectomy showing multiples microcysts with honeycomb aspect. **B** T2-weighted MR image in the axial plane showing typical microcysts in correlation with gross specimen (arrows; Kodama 2). **C** FDG-PET/CT scan showing active parasitic lesion with increased FDG-uptake at the periphery of the lesion. **D** Correlation with diffusion-weighted imaging showing peripheral hypersignal of AE lesion with low ADC values

CT yields better characterization of lesions, in terms of their location, number and size (Fig. 5). An infiltrative mass with calcifications and the absence of contrast enhancement will suggest the diagnosis. CT remains the modality of choice for the detection of calcifications, which may be an important feature of the diagnosis, even non-specific (Table 2). The cystic component within the lesion is variable, and related to the necrotic areas. Peripheral fibrosis may show slight enhancement, on late acquisition. Association with intrahepatic focal dilation of the biliary ducts is frequent, and may occasionally be diffuse in the case of hilar involvement.

**Table 2:** Differential diagnosis for liver calcifications

Differential diagnosis for liver calcifications		Prevalence	Pattern of calcifications	Notes
Parasitic infections	Alveolar echinococcosis	Very common at all stages	Diffuse > Scattered > Focal	Patient from endemic areas Hypoattenuating infiltrative mass, absence of contrast enhancement
	Cystic echinococcosis	May be observed at all stages	Concerns the cyst wall	Patient from endemic areas Circumscribed liver cyst with heterogeneous content (detached membrane or internal cysts)
		CE1: rare	Sprinkled	
		CE2: common	Sprinkled > eggshell-like = circular or content	
		CE3: common	Eggshell-like > sprinkled = circular or content	
		CE4: common	Eggshell-like > sprinkled	
		CE5: very common	Circular or content = eggshell-like > sprinkled Partially or entirely calcified cyst	
Cystic lesions	Cystadenoma/cystadenocarcinoma	8 to 25%	Coarse mural and septal	Cystic lesion with enhanced septa Wall nodule in cystadenocarcinoma
	Liver cysts	Rare	Non-circumferential	Non-enhancing simple cystic lesion
Solid tumors	Cholangiocarcinoma	Rare	Solitary or multiple Ill-defined	Infiltrative mass with delayed enhancement
	Hepatocellular carcinoma (HCC)	Rare (more common after therapy)	Variable	Early enhancement and wash out of solid component in cirrhotic liver
	Fibrolamellar HCC	Very common (40 to 70%)	Calcified central scare	Young adult, large lobulated lesion with central scar in normal liver
	Metastasis	Common in Mucinous carcinoma/osteosarcoma Treated metastasis	Variable	Multiple lesions
	Hemangioma	Rare More frequent if big size	Central and coarse	Peripheral discontinuous enhancement, then centripetal

#### Magnetic resonance imaging (MRI)

MRI provides more precise information concerning the characterization of the lesion, with a heterogeneous infiltrative hypovascular mass that is a mix of solid and cystic tissue. T2-weighted imaging is the most specific sequence, since it enables visualization of metacestodal vesicles (microcysts) and liquefaction necrosis areas (large cysts) in high signal, while granuloma tissue, coagulative necrosis and calcification appear in low signal. Multivesicular lesions on T2-weighted imaging are described as a “bunch of grapes” or “honeycomb” [7] (Fig. 5).

In 2003, Kodama et al. [9] proposed an MRI classification into five types (Table 3). Most AE lesions present microcysts at diagnosis, with a predominance of types 2 and 3, whatever the series (prevalence of 42% for type 2 and 46% for type 3 [9]). As a result, microcysts are the main imaging feature for the positive diagnosis of alveolar echinococcosis.

**Table 3:**
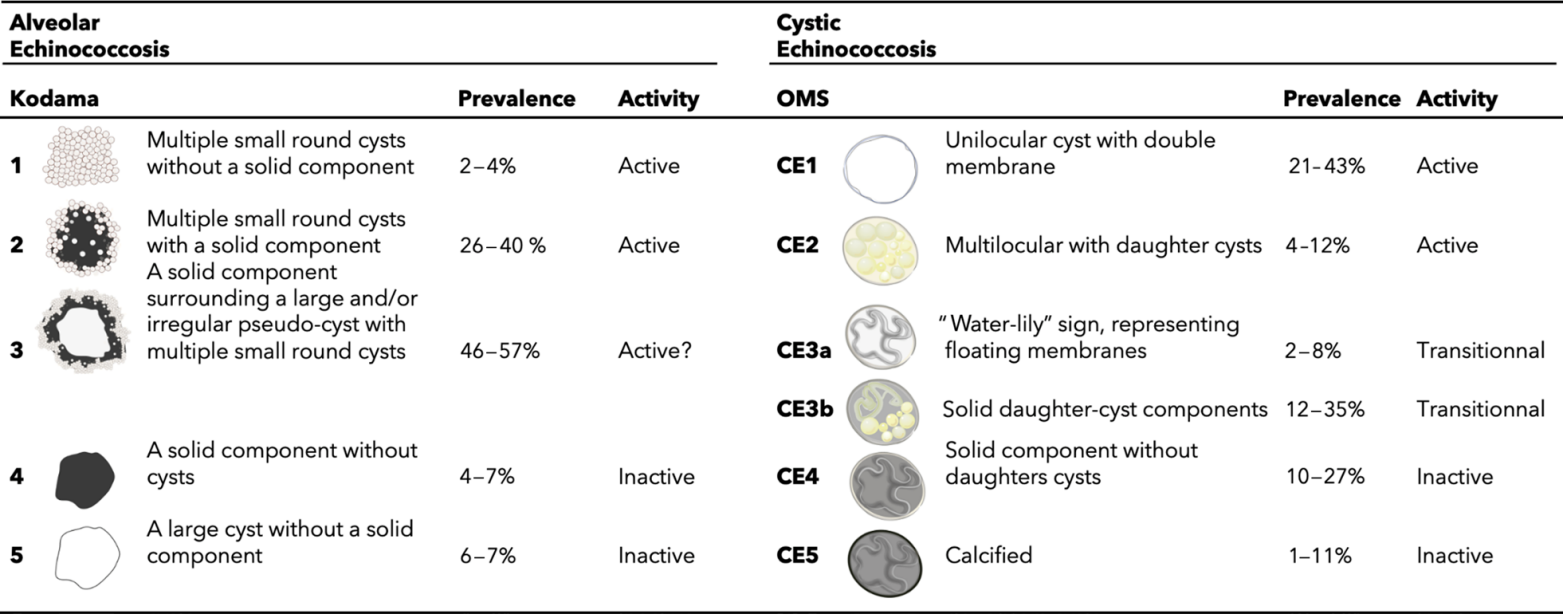
Classifications of AE and CE, and their correlation with parasitic activity and prevalence

**Fig. 6:**
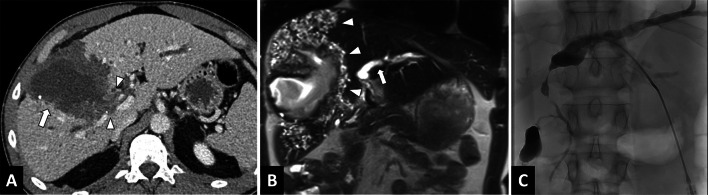
Large alveolar echinococcosis lesion of the right liver in a 34-year-old man diagnosed with jaundice. **A** CT image in the axial plane obtained at portal phase of enhancement showing alveolar echinococcosis of the right liver (arrow) associated with infiltration up to the right pedicle (arrowheads) and the biliary convergence. **B** Coronal T2-weighted MR images. Multiple microcysts (arrowheads) with biliary convergence involvement leading to biliary dilation in the left lobe (arrow). **C** Cholangiography images. Percutaneous biliary drainage to achieve regression of jaundice in preparation for liver surgery

MR cholangiopancreatography (MRCP) is useful for analyzing the potential invasion or compression of the biliary tree by the lesion, in order to guide curative (radical surgical resection) or palliative care (percutaneous or endoscopic interventional procedures) (Fig. 6).

### Serology

AE serology is efficient and is performed either to confirm the diagnosis, when it is suspected on imaging, or to rule out the diagnosis, if there is diagnostic uncertainty [10]. Currently, the serodiagnostic strategy includes primary screening and one or more subsequent confirmatory tests. First-line tests generally consist of EgHF-ELISA or indirect hemagglutination (using *E. granulosus* hydatid fluid) that yields a primary immunodiagnosis for both AE and CE, with a diagnostic sensitivity of 95% for AE. For subsequent specification of the anti-Echinococcus antibody response, *E. multilocularis*-specific ELISA, using the E.m crude antigens (95% specificity) are used. In combination with the Em2-and rec-Em18-ELISA, this immunoblot method makes it possible to diagnose both clinical and sub-clinical (including abortive) AE, with a sensitivity of nearly 100%.

### Role of the radiologist in the diagnosis of AE

Although the diagnosis of AE relies on a combination of the history, serology and imaging, the role of imaging in establishing a positive diagnosis is preponderant (Table 1). If an echogenic liver mass is discovered on US in an endemic area, radiologist should perform CEUS, which will show suggestive findings. Indeed, CEUS of hepatic AE will show absence of internal enhancement in contrast to differential diagnoses such as hemangioma or liver metastases that enhance (Fig. 4).

**Fig. 7:**
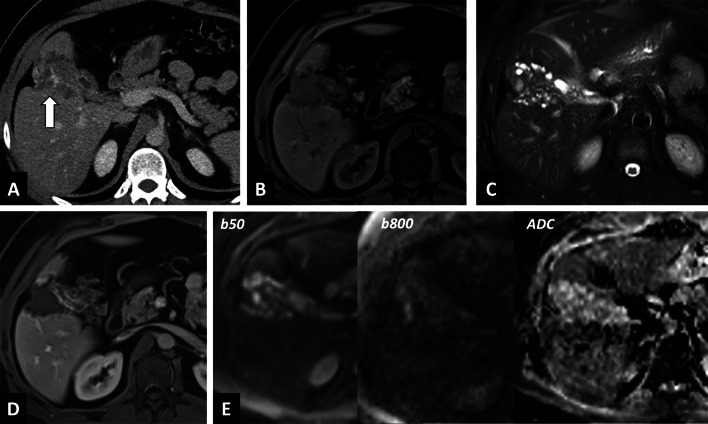
Alveolar echinococcosis in a 57 year old man with an initial suspicion of gallbladder carcinoma. **A** CT images in the axial plane obtained during the portal phase of enhancement: infiltrative hypodense liver lesion with calcifications infiltrating the gallbladder (arrow). **B** Fat-suppressed T1-weighted sequence in the axial plane showing hypointensity of the liver lesion. **C** T2-weighted fat-suppressed MR image in the axial plane showing typical microcysts within hypointense T2 lesions (arrowheads). **D** Diffusion-weighted imaging showing suppressed hyperintensity on b800 corresponding to high ADC values

In case of doubt between an infiltrative mass and an AE lesion, the radiologist should look for microcysts on T2-weighted imaging, which is the pathognomonic elementary lesion of AE. In addition, MRI may show additional features with diffusion-weighted imaging that will help in differentiating AE from cholangiocarcinoma. Increased ADC values will be found in AE lesions, and may be a valuable tool for the differential diagnosis [6] (Fig. 7). CT will be useful in case of an infiltrative AE lesion when microcysts may be difficult to visualize on MRI, to look for calcifications, as for preoperative evaluation.

**Fig. 8:**
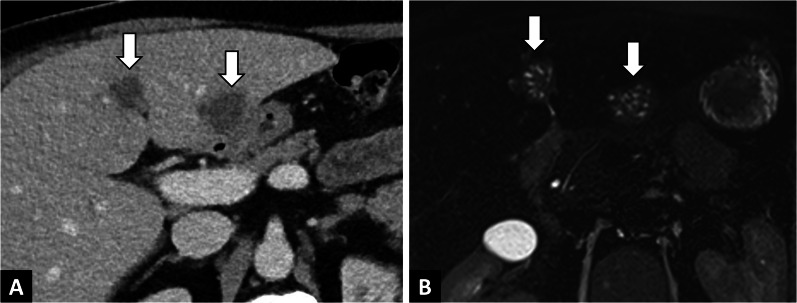
Atypical CT findings of alveolar echinococcosis in a 65-year-old woman with a recent diagnosis of lymphoma. **A** CT image in the axial plane showing two aspecific hypodense liver lesions of the left liver (arrows). **B** T2-weighted fat-suppressed MR image in the axial plane showing typical microcysts within hypointense T2 lesions (arrows; Kodama 2)

The global increase observed in AE cases in endemic areas may be at least partially attributable to the growing number of immunosuppressed human hosts. The rise in cases worldwide has led to increasing reports of atypical imaging findings (i.e., Abscess-, hemangioma-, and metastasis-like) [11, 12] (Fig. 8). Radiologist should be aware of the utility of multimodal imaging in these situations, and may suggest percutaneous biopsy (under ABZ therapy) in this specific population.

### Assessment of parasitic activity

Assessment of parasitic activity is crucial to evaluate possible treatment, but also to monitor the efficacy of antiparasitic treatment. FDG-PET remains the imaging technique of choice for assessment of parasitic activity, since in vitro experimentation in AE lesions has demonstrated high uptake of FDG by immune cells and low uptake by parasitic cells in the vesicles [13]. Consequently, FDG-PET indirectly assesses parasite activity by evaluating the activity of host cells [14]. Delayed acquisition (3 h in addition to the classical 1 h acquisition) is necessary to avoid false negative results, and FDG-PET/CT may detect early hepatic relapse [15].

On MRI, AE activity in the lesions is evaluated by microcyst visualization (Kodama classification 1, 2 and 3) and by DWI. There is a lack of literature data reporting the association between Kodama’s classification and prognosis [3].

**Fig. 9:**
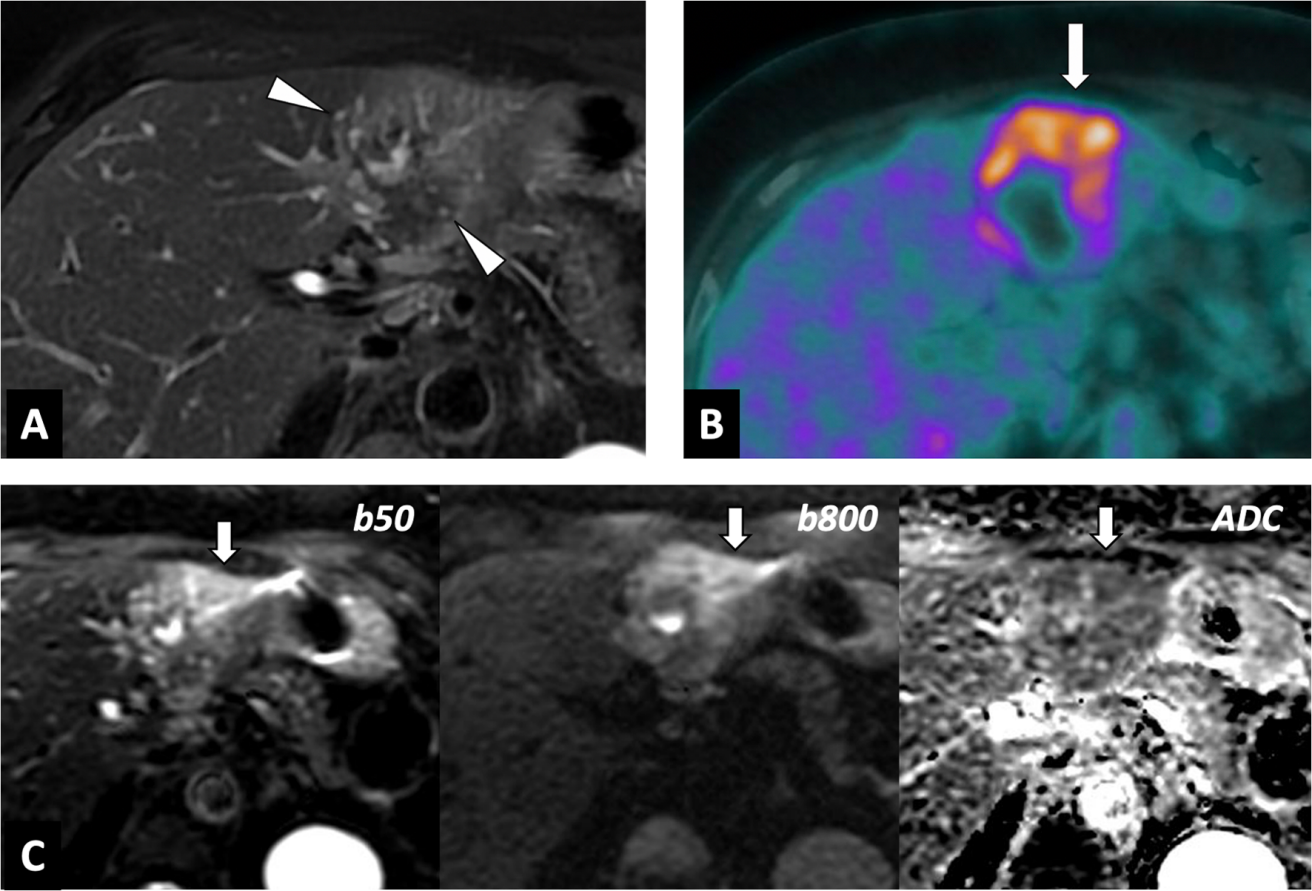
Alveolar echinococcosis lesion of the left liver lobe in 56-year-old woman. Assessment of parasitic activity on MRI and FDG-PET/CT scan. **A** T2-weighted MR image in the axial plane. AE lesion in mixed T2 hypo- and hypersignal, with peripheral microcysts (arrowheads). **B** Diffusion-weighted imaging showing peripheral hypersignal of AE lesion. **C** Correlation with FDG-PET/CT scan. Increased FDG-uptake at the periphery of the lesion

Recent studies [16, 17] have shown that FDG-PET and DWI in MRI provides similar information about the detection of the viability of AE lesions (Figs. 5, 9). In addition, CT may play a role in assessing activity of AE lesions by visualizing microcalcifications [18], which are correlated with hypermetabolic activity on FDG-PET/CT. Further studies are warranted to prospectively assess the potential of FDG-PET/MRI in the management of AE.

### Complications

#### Biliary involvement

**Fig. 10:**
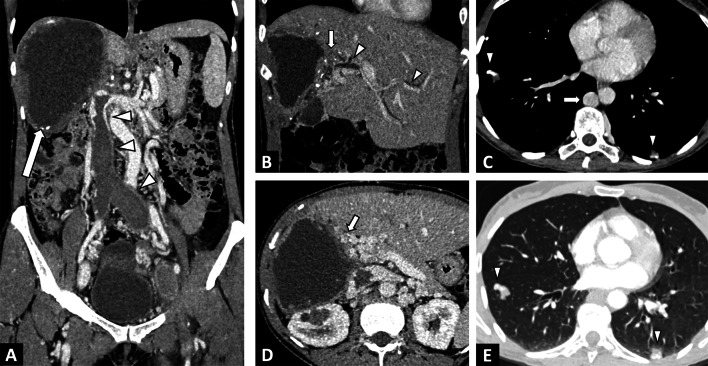
Alveolar echinococcosis lesion of the right liver in a 49-year-old woman discovered due to left leg edema. **A** CT image in the coronal plane at the portal phase showing alveolar echinococcosis of the right liver (arrow) associated with invasion of the inferior vena cava, responsible for thrombosis, extending to the common iliac veins (arrowheads). **B** CT image in the coronal plane obtained during the portal phase: infiltration to the pedicle and biliary convergence (arrows) and biliary dilatation of the left lobe (arrowheads). **C** CT image in the axial plane obtained during the portal phase showing portal cavernoma of the liver pedicle secondary to hilar alveolar echinococcosis infiltration. **D** Same examination, soft tissue window: calcified lung nodules (arrowheads). Hypertrophy of the azygos vein secondary to inferior vena cava thrombosis (arrow). **E** Same examination, lung window: calcified lung nodules (arrowheads)

Biliary involvement is the leading complication of AE, and is mainly revealed by jaundice or cholangitis (Figs. 6, 10). AE infiltrates the hilum, the vascular structures and the proximal biliary ducts. Overall prevalence of biliary complications is about 10% [19, 20] and 30% in case of non-resectable AE [21]. Biliary complications can be observed at diagnosis or may appear during follow-up. In these situations, percutaneous biliary drainage may be envisaged if endoscopic drainage is impossible.

#### Vascular complication

**Fig. 11:**
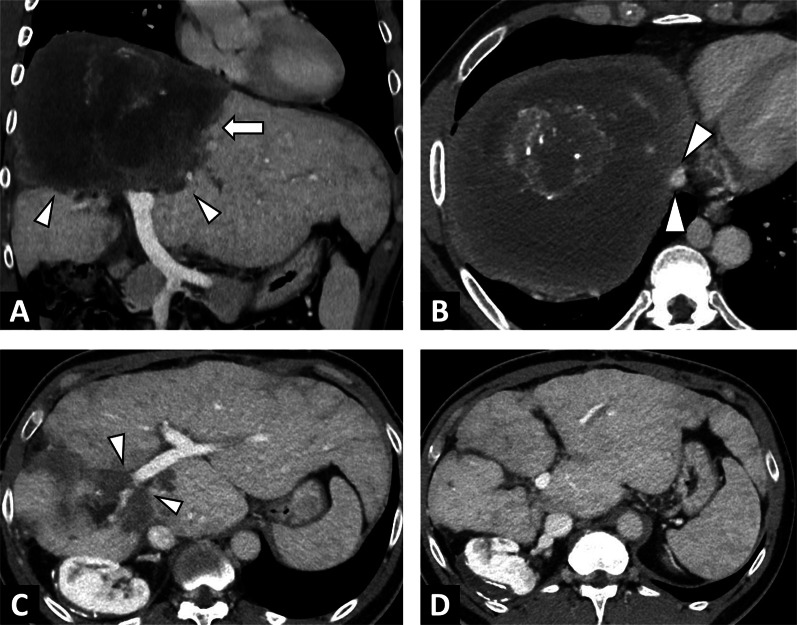
Alveolar echinococcosis lesion of the right liver in a 44-year-old man complicated by advanced chronic liver disease secondary to Budd Chiari syndrome. **A** CT image in the coronal plane at the portal phase showing alveolar echinococcosis of the right liver (arrow) associated with infiltration to the pedicle (arrowheads). **B** CT image in the axial plane obtained during the portal phase: invasion of the inferior vena cava and the ostium of the hepatic veins (arrowheads). **C** CT image in the axial plane obtained during the portal phase: infiltration of the right liver pedicle (arrowheads) D. Hypertrophy of the left liver lobe and surface nodularity suggestive of advanced fibrosis. Note the mosaic pattern of enhancement, sign of hepatic sinusoidal dilatation, as a result of the Budd Chari syndrome

**Fig. 12:**
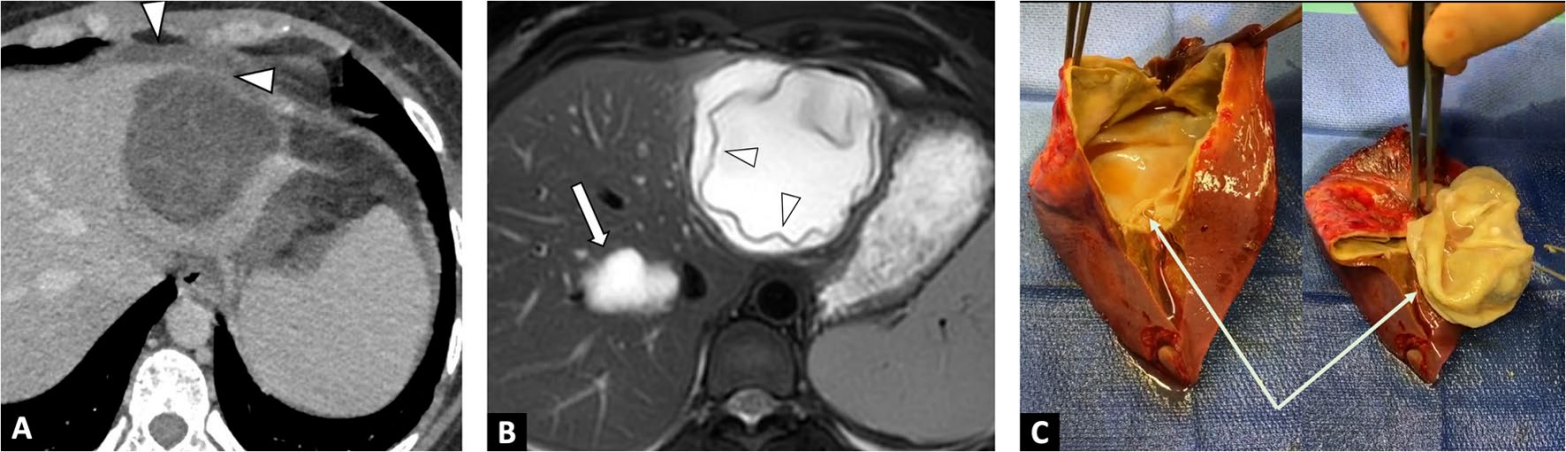
Left lobe cystic echinococcosis lesion in a 43-year-old woman revealed by abdominal pain associated with fever. **A** CT image in the axial plane obtained during the portal phase of enhancement showing peritoneal rupture of left lobe CE lesion (arrowheads). Peritoneal effusion contiguous to the anterior surface of the left lobe CE lesion. **B** T2-weighted fat-suppressed MR image in the axial plane showed internal detached membrane (water lily sign, arrowheads) of the left lobe lesion, diagnosing a transitional stage of CE (CE3a). Second CE lesion CE1 (arrow). **C** Correlation with macroscopic findings. Internal membrane of the CE3a cyst (arrows)

As AE is infiltrative, it can invade the major hepatic veins and retro-hepatic inferior vena cava (Fig. 11), which in turn can lead to complications ranging from simple sinusoidal dilatation and intrahepatic venous shunt to Budd-Chiari syndrome [22–24] (Fig. 12). In these situations, as surgical treatment will require large hepatic resections, evaluation of liver fibrosis will be crucial. Noninvasive techniques (liver surface nodularity at CT, US elastography by Shearwave) are helpful, although liver biopsy may often be required. Cross-sectional imaging should look for imaging features of sinusoidal dilation, and infiltration of major hepatic veins.

#### Extra-hepatic involvement

Extra-hepatic involvement remains rare in AE, and seems to be more frequent in China than in Europe [25], probably due to the fact that cases from China are often more advanced at the time of diagnosis. The most common site of extrahepatic manifestation in AE is the lung, followed by the brain, which explains why chest CT and brain MRI are included in the work-up of AE patients. Lung AE metastasis will appear as nodules, frequently calcified (Fig. 9). Less frequently, AE may involve the peritoneum [26] or vertebrae [27]. Of note, a significant relationship exists between the presence of distant extrahepatic disease and the size of the liver lesion [25].

### Treatment

Whatever the situation, benzimidazole (mainly ABZ) is the common denominator for all the therapeutic options in AE, either to support curative surgical treatment or in the long term, if surgical treatment is not possible.

Curative liver resection remains the gold standard treatment in AE, and the only one that may lead to definitive cure. However, since AE lesions are most often located in the right lobe of the liver, and in advanced cases, may have invaded the major bile ducts and vessels (portal veins, hepatic veins, and vena cava), major hepatic surgery is often required. Consequently, preoperative imaging is crucial. Triple phase CT scan (unenhanced acquisition, late arterial and portal phase) remains the imaging technique of choice for preoperative hepatic AE because of the spatial resolution it provide. This assessment should focus on identifying the slightest contact with structures such as hepatic venous confluence, inferior vena cava or diaphragm and hilar infiltration should be suspected when parasitic infiltration is close [28]. In the same way as for infiltrating cancers such as hilar cholangiocarcinoma or pancreatic adenocarcinoma, multidisciplinary management of surgical cases is crucial, and should involve preoperative discussions between radiologists and surgeons.

For patients not amenable to surgery, long-term ABZ therapy should be prescribed. Massively calcified or metabolically inactive AE lesions are generally only monitored (“watch and wait” strategy) [5]. Liver allotransplantation is the last resort in advanced cases, especially in case of incurable symptomatic vascular or biliary complications [5].

Interventional radiology may be involved in preoperative management of AE lesions with portal embolization to enlarge future remnant liver volume, or in the management of complications (palliative treatment). Percutaneous drainage to evacuate an infected necrotic cavity, and percutaneous transhepatic biliary drainage to treat bile duct obstruction may be necessary in rare situations (Fig. 6). Contrary to EC, there is no particular risk of anaphylactic reaction in case of percutaneous drainage of alveolar echinococcosis.

### Follow-up

For patients treated by curative surgery, long-term follow-up by US at shorter intervals and CT and/or MRI at intervals of 2–3 years should be planned due to the risk of recurrence, even after several years of remission [29]. The combination of FDG-PET with anti-EmII/3–10 and more recently Em18 antibody levels seems to be the best association to evaluate AE activity after long-term ABZ therapy for non-resectable AE [30]**.**

## Cystic echinococcosis (CE)

Cystic echinococcosis (CE), also known as hydatidosis or hydatid disease, is a widely endemic disease caused by metacestode of the *Echinococcus granulosus* tapeworm. CE is responsible for a wide spectrum of manifestations, ranging from asymptomatic cases to fatal infection. Unlike AE, CE is mostly represented by one large cyst, from which complications arise, predominantly spontaneous rupture.

### Physiopathology

*Echinococcus granulosu*s is present on every continent except the Antarctic and is endemic in South America, Eastern and Southern Europe, Russia, the Middle East, Africa and China. Humans are accidentally contaminated by dogs in livestock-raising areas. Just as in AE, the parasite reaches the liver by the same pathophysiological mechanism [5]. Unlike AE, however, the metacestode forms a cyst that grows slowly, and can remain asymptomatic for 10–15 years. It becomes symptomatic when it reaches 10 cm in diameter or in case of complications. The natural history of hydatid disease in humans is not fully known. Indeed, only very fragmentary data are available from mass US screening in endemic countries with longitudinal follow-up of variable duration; the numbers are often small. Figure 3 shows a schematic structure of cystic echinococcosis, with pericyst (the inflammatory reaction of the host, progressively leading to the constitution of a fibrous shell within which calcic deposits may appear over time), in contrast to the hydatid component (laminated and germinated layer) [31].

### Imaging findings

The diagnosis is based on history, clinical examination, serology and imaging. The imaging diagnosis will be easy when evidence of the internal membrane or daughter cyst is visualized, but challenging in case of a simple cyst with parietal calcifications.

#### Ultrasound

US remains the imaging technique of choice for stage diagnosis of CE lesions. It makes it possible visualize the matrix within the cyst, and enables detection of calcifications. US of CE shows a liver cyst with an echogenic component. Visualization of an internal detached membrane, and internal anechogenic cysts will be the key point for the CE diagnosis. The World Health Organization Working Group on Echinococcosis (WHO-IWGE) international classification is based on US imaging (Table 3), whereby types CE1/CE2 correspond to the “active stages,” i.e., CE1 unilocular cyst and CE2 multilocular with daughter cysts; and types CE4/CE5 correspond to “degenerating stages.” These two latter types, frequently non-viable (CE4 and CE5), show an echogenic lesion with calcification and fibrosis. Types CE3a and CE3b correspond to “transitional stages” and are subdivided by morphological criteria. CE3a is characterized by the “water-lily” sign, representing floating membranes (Fig. 12), while CE3b is characterized predominantly by solid daughter-cyst components.

#### MRI

MRI, especially with T2-weighted images, provides good diagnostic performance for internal matrix visualization but can have shortcomings in identifying details of the cyst wall [32]. It remains the best imaging modality for evaluating large cysts, as it provides an overall view of the cysts, and can visualize all cyst components (CE2 to CE5). Furthermore, MRI remains the best imaging technique to detect biliary fistula using MRCP sequences [33]. After contrast injection, T1-weighted imaging will show a total absence of enhancement by the content of the cystic lesion.

#### CT

**Fig. 13:**
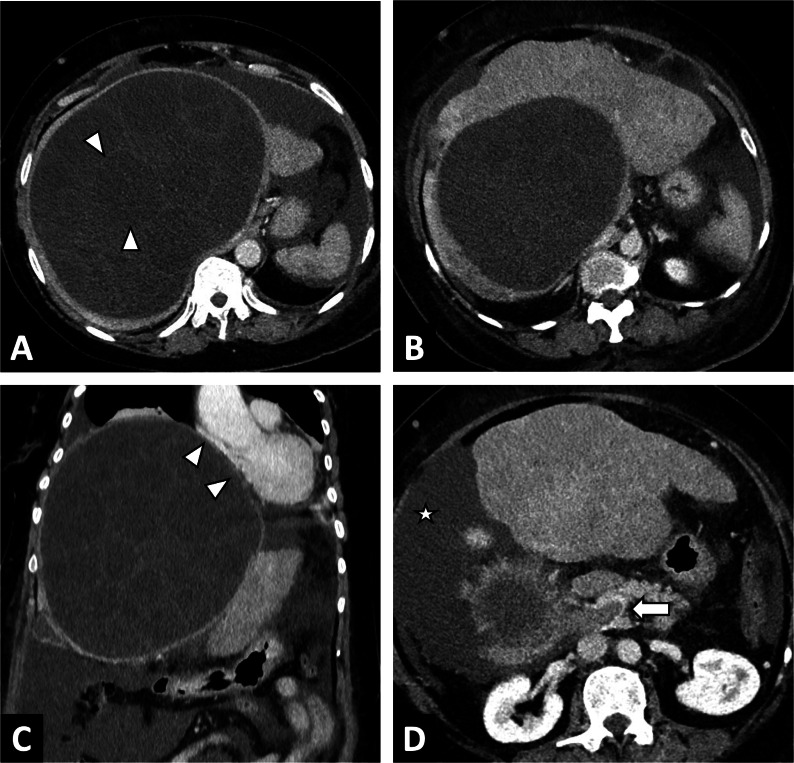
Large cystic echinococcosis lesion of the right liver in a 54-year-old woman revealed by ascitic decompensation. **A** CT image in the axial plane at the portal phase showing a large cystic echnicoccosis lesion of the right liver. Internal daughter cysts (arrowheads) (CE3a stage). **B** CT image in the axial plane at the portal phase showing a large right liver cyst complicated by advanced chronic liver disease secondary to Budd Chiari syndrome. Hypertrophy of the left liver lobe and surface nodularity suggestive of advanced fibrosis. **C** Same examination in the coronal plane. Mass effect of the cystic echinococcosis on the heart chambers (arrowheads). **D** Same examination in the axial plane, lower slices. Portal thrombosis (arrow) and ascites (star)

CT is not the first line imaging modality, but is frequently the means by which CE is discovered. It can be challenging in CE1 to CE4 lesions, as it does not enable precise visualization of the cyst matrix [32], but is a good imaging technique for visualizing calcifications. Calcifications of the cyst wall may occur at all stages and are not restricted to the inactive CE4 or CE5 types [34]. Indeed, although the prevalence of calcification increases with progression of the cyst degenerative process, it is not synonymous with parasite inactivity [34]. From a practical point of view, if a CE with a calcified wall is observed on CT, the evaluation of the cyst contents on imaging remains crucial to determine its activity, by US imaging or MRI, depending on the extent of the calcifications. Finally, CT will be a more important imaging approach for detecting CE complications [35] (Fig. 13).

### Serology

Contrary to AE, specific serology suffers from a lack of sensitivity and plays only a secondary role in the diagnosis of CE. The first line tests available are the same as for AE (indirect hemagglutination, ELISA with E. g antigens). These first-line serology tests can be negative in 30–58% of cases for CE1 cysts, and in more than half of cases for inactive cysts CE4/CE5 [36]. The highest sensitivity is observed for CE2 and CE3 stages. The confirmatory test (Western Blot) is more sensitive and may be performed if the first line tests are negative, when the epidemiological, clinical and imaging context pleads in favor of a diagnosis of CE.

### Role of radiologist in CE diagnosis

Radiologists will frequently be involved in ascertaining the CE diagnosis. In endemic countries, US is considered as the first line imaging approach, as it is widely available and enables good visualization of the matrix cyst. In case of a fortuitous discovery on US imaging, the radiologist must discuss the CE lesion in case of a cystic liver lesion showing heterogenous contents, including a detached membrane or internal anechogenic cysts. Radiologists will have to discuss the CE diagnosis more largely, in case of patients coming from endemic areas. Therefore, the radiologist must have good knowledge of the markers of parasitic activity (internal cysts, detached membrane) and must be aware that incomplete exploration on ultrasound will have to be combined with MRI findings, and vice versa. This is all the more important since, in CE, the serology can be negative in up to 58% of cases with CE1 cysts, in more than half of cases for inactive CE4/CE5 cysts, and in 5–20% of CE2 and CE3 cysts [36].

**Fig. 14:**
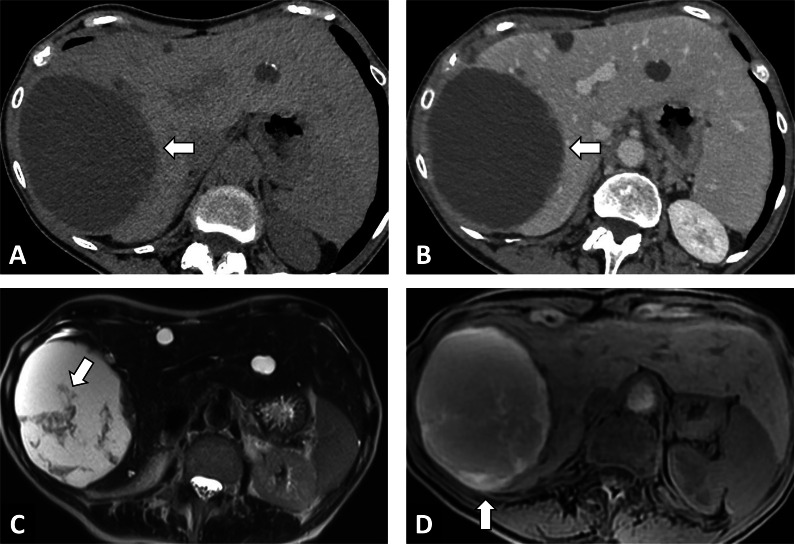
Differential diagnosis of a cystic echinococcosis lesion in a 78-year-old woman. **A** Unenhanced CT image in the axial plane showing a right liver cyst. **B** CT image in the axial plane at the portal phase showing a cystic lesion of the right liver with thickening wall. **C** T2-weighted MR image in the axial plane showing heterogeneous content without daughter cysts. **D** T1-weighted MR image in the axial plane showing hyperintense signal of the liver cyst confirming the diagnosis of hemorrhagic liver cyst

**Fig. 15:**
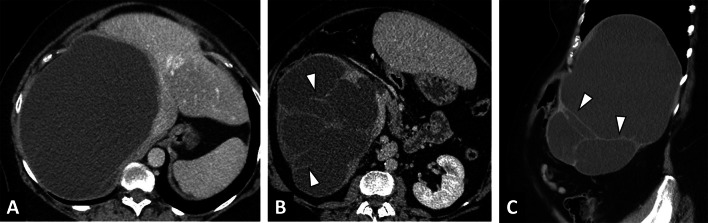
Differential diagnosis of cystic echinococcosis lesion in a 64-year-old woman. **A** CT image in the axial plane at the portal phase showing a large right liver cyst. **B** Same examination, lower slices. Internal enhanced septa. **C** CT image in the sagittal plane at the portal phase showing internal enhanced septa ruled out a cystic echinococcosis lesion, confirming the diagnosis of biliary mucinous cystadenoma

The main differential diagnosis of CE will be a liver cystic lesion associated with calcifications (Table 2, Fig. 14). Consequently, the radiologist must look for imaging features that will rule out the CE diagnosis, mainly internal enhanced septa (Fig. 15), as well as specific imaging features of CE (daughter cysts and internal detached membrane).

### Activity assessment

**Fig. 16:**
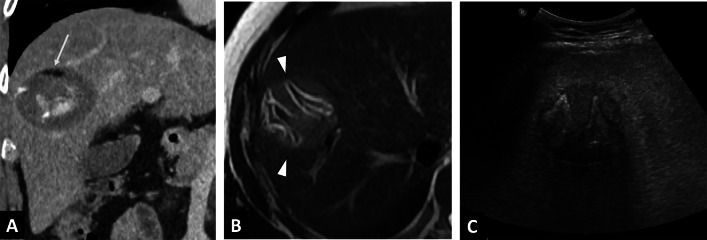
Cystic echinococcosis lesion CE4 with negative result on serology test. **A** CT image in the coronal plane obtained during the portal phase of enhancement: CE lesion of the right lobe with calcified membrane, leads to complementary exploration in MRI and US to assess activity. Note the macroscopic fat content (arrow). **B** T2-weighted MR image in the axial plane showing low signal in T2 and absence of daughter cysts (arrowheads). **C** B-mode ultrasound: echogenic structure of the CE lesion, without daughter cyst. Lesion classified as CE4

Activity assessment of CE lesions is simpler than in AE. The WHO-IWGE classification is correlated to parasitic activity [37] and imaging aims to detect internal cysts or internal detached membranes. In case of a doubtful matrix cyst, the radiologist must switch to another imaging technique, e.g., add MRI if US shows extensive parietal calcifications, and try US if MRI shows a doubtful matrix between CE3 and CE4 (Fig. 15). Evidence of fat content (CT or MRI) within cystic echinococcosis lesions can be observed in rare cases. While some authors have described large fat content as a result of biliary communication [38], others have described small foci of fat content within the cyst as a sign of a degenerating cyst, CE4 to CE5 (Fig. 16) [39].

### Complications

CE is often asymptomatic, but symptoms occur largely when complications develop. The three main complications of CE are mechanical complications (rupture of the cyst or compression), secondary infection and anaphylactic reaction. Risk factors for complications are difficult to evaluate since a large proportion of cysts are asymptomatic and undiagnosed. Nevertheless seem to be more frequent in younger patients, whereas the link with cyst size is not clear [40].

#### Mechanical complications

**Fig. 17:**
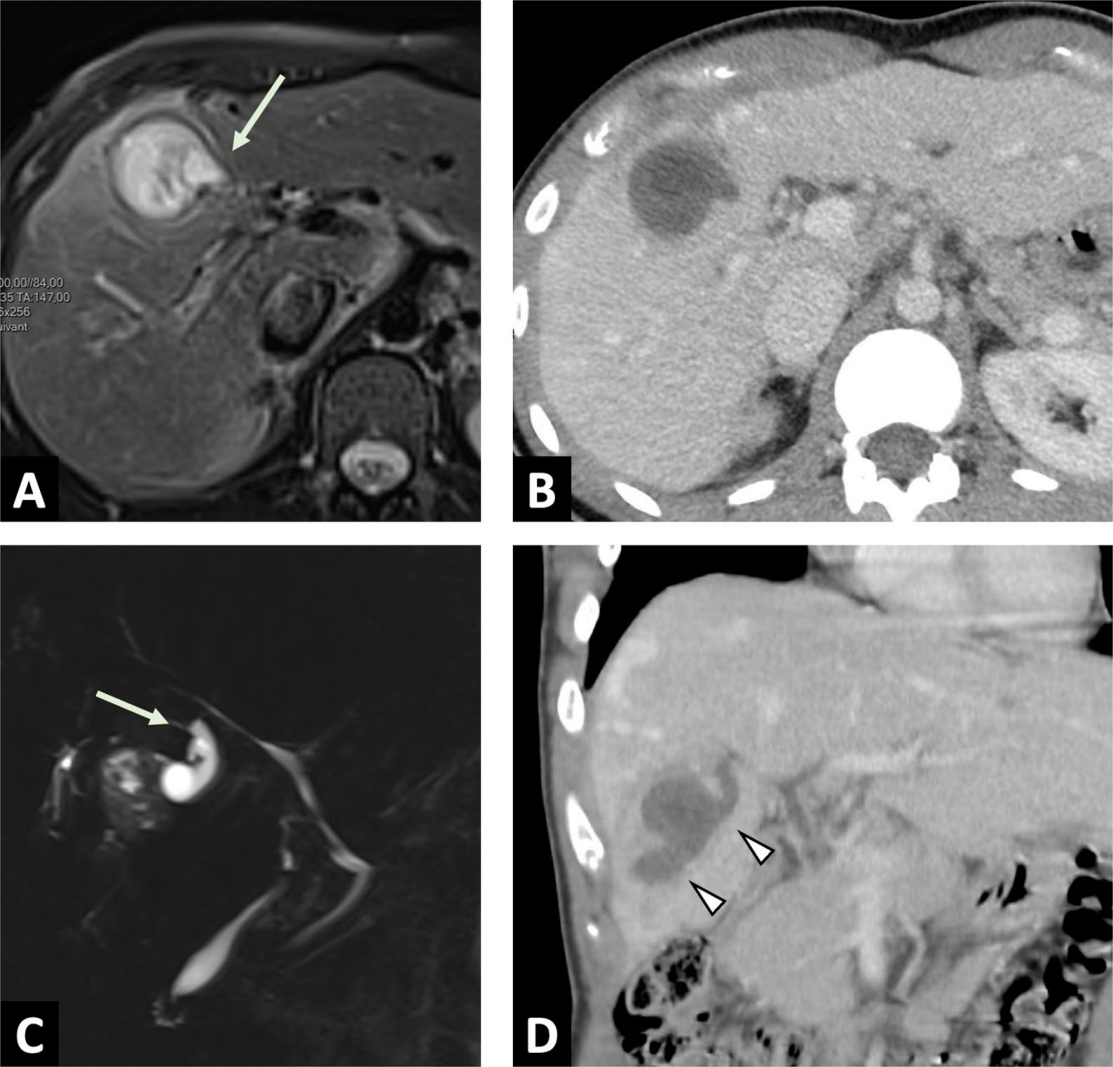
Cystic echinococcosis lesion CE3b of the right liver lobe in a 44-year-old man. **A** T2-weighted MR image in the axial plane: loss of continuity of the cyst wall and visualization of daughter cyst into the biliary duct (arrow). **B** Corresponding images on CT obtained during the portal phase of enhancement. **C** MRCP in the coronal plane: direct communication between the cyst and the biliary duct (arrow). **D** Corresponding images on CT obtained during the portal phase of enhancement: direct communication between the cyst and the biliary duct (arrowheads)

Macroscopic rupture in CE and secondary fistula are reported as the main complications of CE [41, 42]. Rupture of CE within the biliary tree is the main mechanical complication (Fig. 17). This is a serious event, since biliary obstruction by hydatid debris can lead to pancreatitis, cholangitis or septicemia.

The main US feature for diagnosing intrabiliary rupture of a hydatid cyst [43] is a loss of continuity of the cyst wall in contact with an adjacent bile duct, which is a direct and pathognomonic sign, but rarely observed. Cystic images in the bile ducts are very characteristic of rupture but not frequently observed. Linear hyperechogenic images and non-shadowing material can be found, or dilation of the biliary tree and thickening of the bile ducts are signs of cholangitis.

**Fig. 18:**
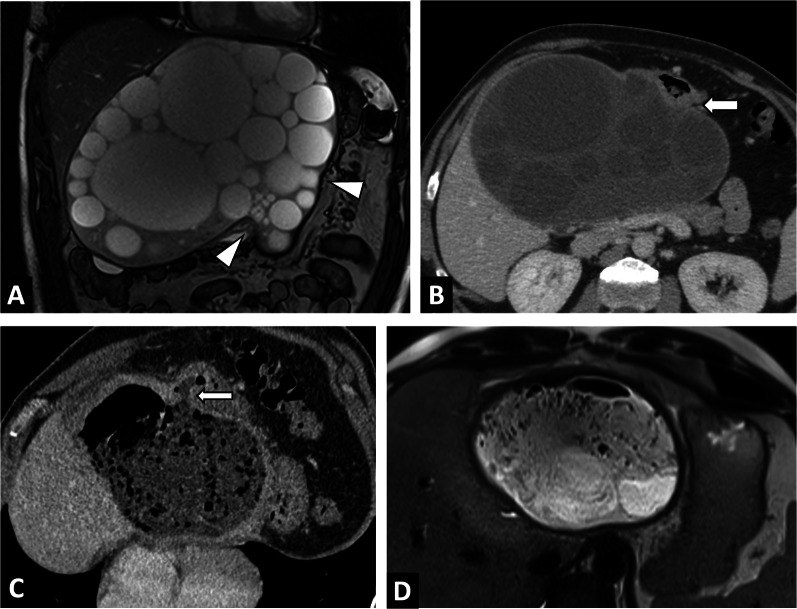
Large cystic echinococcosis lesion of the left liver at CE3b stage discovered during an investigation of abdominal pain. **A** T2-weighted MR image in the coronal plane: loss of continuity of the cyst wall (arrowheads) in contact with the gastric antrum. **B** CT image in the axial plane obtained during the portal phase of enhancement: close contact between the cyst wall and the gastric antrum (arrow). **C** CT image in the axial plane obtained during the portal phase in a context of fever and abdominal pain 3 months after the start of anti-parasitic treatment. Appearance of digestive content and air-fluid level within the CE lesion. Direct communication between the cyst and the gastric antrum (arrow). **D** Same lesion in T2-weighted MR image in the axial plane. Note the emptying of most daughter cysts, a sign of evacuation in the digestive tract

**Fig. 19:**
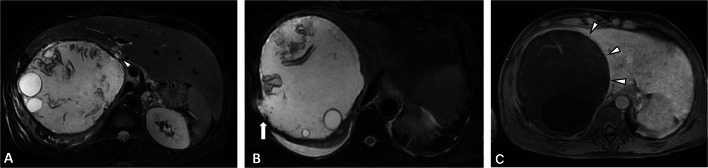
Cystic echinococcosis lesion CE3b in a 48-year-old patient revealed by abdominal pain, fever, and liver blood test abnormalities. **A** Fat-suppressed T2-weighted MR image in the axial plane shows a cystic lesion of the right liver, including daughter cysts with floating membranes. Biliary compression without fistula can be observed (arrow). **B** Fat-suppressed T2-weighted MR image in the axial plane. Posterior rupture of the cystic echinococcosis (arrow). **C** Fat-suppressed T1-weighted sequence in the axial plane shown after gadolinium injection at the portal phase. Parietal enhancement of cystic echinococcosis that suggests infection of parasitic lesion (arrowheads)

Intraperitoneal rupture can occur when CE is peripheral or located in the left liver lobe (Fig. 18). It should be suspected if CE lesions are peripheral and associated with peritoneal fluid. In acute symptomatic rupture, peritoneal irritation, acute abdominal symptoms (Fig. 19) and allergic reactions will occur and patient may develop disseminated peritoneal CE.

CE lesions can also compress the bile ducts, portal veins, hepatic veins and inferior vena cava and can cause portal hypertension [44] and Budd Chiari syndrome (Fig. 13).

#### Infectious complications

The development of biliary cystic communication leads to small amount of hydatid fluid within the biliary tree, which is a prerequisite for bacterial contamination. Bacterial contamination leads to the formation of abscesses with the same US appearance as other types of liver abscess: poor delimitation, heterogeneous echostructure and intracystic air-fluid, or fluid–fluid levels [45]. A peripheral hypervascular area can be found by contrast-enhanced CT and MRI [45] (Fig. 19).

#### Extrahepatic involvement

Contrary to AE, CE is frequently characterized by extrahepatic involvement. The other organs affected are mainly the lung (25–40% of cases [46–48]), which should be explored if hydatid disease is diagnosed. In addition, various other locations may be observed (spleen, brain, kidneys adrenal glands, bones). Imaging features of extrahepatic CE will be usually the same as in liver CE, i.e., cystic lesions with internal cysts or detached membranes.

### Treatment

As in AE, the role of imaging is essential in the therapeutic management of hydatid cyst, and depends on the WHO-IWGE classification, location, size and the presence of complications.

Anti-infectious treatment is based on continuous administration of ABZ. It may sometimes be given life-long in multiple, inoperable cysts or for a fixed duration in association with potentially curative surgical or instrumental therapeutic options. The only curative treatment is complete surgical removal of the cyst. Total cystectomy, which avoids opening the cyst, is the technique of choice [1, 29].

Percutaneous treatment by Puncture-Aspiration of cyst contents-Injection of protoscolecidal agents-Reaspiration (PAIR) has been largely developed in endemic countries [49]. A more recent technique, the modified catheterization technique (MoCAT), consisting in first aspirating the cyst contents including the parasitic membranes, then leaving the catheter in place temporarily after the procedure [50] is currently under evaluation for large cysts with multiple daughter cysts and/or partially solid matrix content. Regardless of the technique used, communication with the biliary tree should be carefully investigated, given the risk of cholangitis induced by the scolicidal agents. Insufficient destruction of the germinal membranes or protoscoles during percutaneous procedures, or peri-operative dissemination are the main causes of recurrence. ABZ therapy should be given in association with interventional techniques (PAIR and MoCAT), and surgery (5). Finally, anaphylaxis during PAIR procedures should be anticipated by steroid and antihistaminic premedication and ready availability of adrenaline [49].

For inactive cysts (CE4 and CE5), a “Watch and Wait” approach is recommended (20). ABZ alone may be efficient for small CE1 and CE3a cysts (< 5 cm). Large CE1 and CE3a cysts (> 5 cm) can be treated either with PAIR or with surgery, depending on their location in the liver, and local expertise. Currently, the preferred option for CE2 and CE3b lesions is surgery, but the MoCAT technique is still under evaluation for these stages.

### Follow-up

It is widely accepted that close biological and imaging follow-up for at least 5 years is necessary in CE because of the risk of recurrence after surgical treatment and the uncertain results of other techniques. In addition, biological follow-up (liver enzymes and blood cell count) during the first 6 months is necessary to check for possible ABZ toxicity [5].

## Conclusion

In conclusion, the two tapeworms of the Echinococcus genus cause liver diseases that share some similarities in terms of diagnostic approach and treatment, but each associated with specific imaging findings. Radiologists should be aware of these two forms of parasitic liver infection, as it will be necessary to discuss imaging findings in patients with echinococcosis, and they will have to be referred for specific examinations for the assessment of parasitic activity. Radiologists should also be aware of the role of interventional radiology in these affections, as it may be useful for the treatment of certain complications in AE or for treating certain forms of CE. Multidisciplinary teams are crucial for echinococcosis management, and radiologists have an important role to play in these teams, given the central role of imaging in these two diseases.

## Data Availability

Not applicable.

## Abbreviations

ABZ: Albendazole
AE: Alveolar echinococcosis
CE: Cystic echinococcosis
CEUS: Contrast-enhanced ultrasound
CT: Computed tomography
DWI: Diffusion-weighted imaging
FDG-PET: Fluorine-18-fluorodeoxyglucose positron emission tomography
MoCAT: Modified catheterization technique
MRCP: Magnetic resonance cholangiopancreatography
MRI: Magnetic resonance imaging
PAIR: Puncture-aspiration-injection-reaspiration
US: Ultrasound
WHO-IWG: World Health Organization Working Group on Echinococcosis

## References

[1] McManus DP, Zhang W, Li J, Bartley PB (2003). Echinococcosis. Lancet.

[2] Eckert J, Deplazes P (2004). Biological, epidemiological, and clinical aspects of echinococcosis, a zoonosis of increasing concern. Clin Microbiol Rev.

[3] Bulakçı M, Kartal MG, Yılmaz S (2016). Multimodality imaging in diagnosis and management of alveolar echinococcosis: an update. Diagn Interv Radiol.

[4] Bresson-Hadni S, Delabrousse E, Blagosklonov O (2006). Imaging aspects and non-surgical interventional treatment in human alveolar echinococcosis. Parasitol Int.

[5] Wen H, Vuitton L, Tuxun T (2019). Echinococcosis: advances in the 21st century. Clin Microbiol Rev.

[6] Kantarci M, Bayraktutan U, Karabulut N (2012). Alveolar echinococcosis: spectrum of findings at cross-sectional imaging. Radiographics.

[7] Liu W, Delabrousse É, Blagosklonov O (2014). Innovation in hepatic alveolar echinococcosis imaging: best use of old tools, and necessary evaluation of new ones. Parasite.

[8] Li J, Dong J, Yang L, Li X, Song T (2018). Comparison of [18F]fluorodeoxyglucose positron emission tomography and contrast-enhanced ultrasound for evaluation of hepatic alveolar echinococcosis activity. Ultrasound Med Biol.

[9] Kodama Y, Fujita N, Shimizu T (2003). Alveolar echinococcosis: MR findings in the liver. Radiology.

[10] Bresson-Hadni S, Spahr L, Chappuis F (2021). Hepatic alveolar echinococcosis. Semin Liver Dis.

[11] Weiner SM, Krenn V, Koelbel C, Hoffmann HG, Hinkeldey K, Ockert D (2011). *Echinococcus multilocularis* infection and TNF inhibitor treatment in a patient with rheumatoid arthritis. Rheumatol Int.

[12] Chauchet A, Grenouillet F, Knapp J (2014). Increased incidence and characteristics of alveolar echinococcosis in patients with immunosuppression-associated conditions. Clin Infect Dis.

[13] Reuter S, Buck A, Manfras B (2004). Structured treatment interruption in patients with alveolar echinococcosis. Hepatology.

[14] Porot C, Knapp J, Wang J, et al (2014) Development of a specific tracer for metabolic imaging of alveolar echinococcosis: a preclinical study. In: 2014 36th annual international conference of the IEEE engineering in medicine and biology society, 2014, pp 5587–5590.10.1109/EMBC.2014.6944893 10.1109/EMBC.2014.6944893 25571261

[15] Caoduro C, Porot C, Vuitton DA (2013). The role of delayed 18F-FDG PET imaging in the follow-up of patients with alveolar echinococcosis. J Nucl Med.

[16] Lötsch F, Waneck F, Groger M (2019). FDG-PET/MRI imaging for the management of alveolar echinococcosis: initial clinical experience at a reference centre in Austria. Trop Med Int Health.

[17] Zheng J, Wang J, Zhao J, Meng X (2018). Diffusion-weighted MRI for the initial viability evaluation of parasites in hepatic alveolar Echinococcosis: comparison with positron emission tomography. Korean J Radiol.

[18] Brumpt E, Blagosklonov O, Calame P, Bresson-Hadni S, Vuitton DA, Delabrousse E (2019). AE hepatic lesions: correlation between calcifications at CT and FDG-PET/CT metabolic activity. Infection.

[19] Graeter T, Ehing F, Oeztuerk S (2015). Hepatobiliary complications of alveolar echinococcosis: a long-term follow-up study. World J Gastroenterol.

[20] Ozturk G, Polat KY, Yildirgan MI, Aydinli B, Atamanalp SS, Aydin U (2009). Endoscopic retrograde cholangiopancreatography in hepatic alveolar echinococcosis. J Gastroenterol Hepatol.

[21] Frei P, Misselwitz B, Prakash MK (2014). Late biliary complications in human alveolar echinococcosis are associated with high mortality. World J Gastroenterol.

[22] Soyer V, Ara C, Yaylak F (2015). Advanced alveolar echinococcosis disease associated with Budd–Chiari syndrome. Int J Surg Case Rep.

[23] Kobryń K, Paluszkiewicz R, Dudek K (2017). Good outcome following liver transplantation using pericardial-peritoneum window for hepato-atrial anastomosis to overcome advanced hepatic alveolar echinococcosis and secondary Budd–Chiari Syndrome: a case report. BMC Surg.

[24] Çakmak E, Alagozlu H, Gumus C, Alí C (2013). A case of Budd–Chiari syndrome associated with alveolar echinococcosis. Korean J Parasitol.

[25] Graeter T, Bao H-H, Shi R (2020). Evaluation of intrahepatic manifestation and distant extrahepatic disease in alveolar echinococcosis. World J Gastroenterol.

[26] Beaussant-Cohen S, Richou C, Lenoir M, Grenouillet F, Bresson-Hadni S, Delabrousse E (2018). MR imaging features of peritoneal alveolar echinococcosis. Diagn Interv Imaging.

[27] Meinel TR, Gottstein B, Geib V (2018). Vertebral alveolar echinococcosis-a case report, systematic analysis, and review of the literature. Lancet Infect Dis.

[28] Calame P, Doussot A, Turco C, Colpart P, Heyd B, Delabrousse E (2021). Local invasion of hepatic alveolar echinococcosis should not be underestimated: lessons learned from imaging-pathologic correlation. Diagn Interv Imaging.

[29] Brunetti E, Kern P, Vuitton DA, Writing Panel for the WHO-IWGE (2010). Expert consensus for the diagnosis and treatment of cystic and alveolar echinococcosis in humans. Acta Trop.

[30] Ammann RW, Stumpe KDM, Grimm F (2015). Outcome after discontinuing long-term benzimidazole treatment in 11 patients with non-resectable alveolar echinococcosis with negative FDG-PET/CT and Anti-EmII/3-10 serology. PLoS Negl Trop Dis.

[31] Vuitton DA, McManus DP, Rogan MT (2020). International consensus on terminology to be used in the field of echinococcoses. Parasite.

[32] Stojkovic M, Rosenberger K, Kauczor H-U, Junghanss T, Hosch W (2012). Diagnosing and staging of cystic echinococcosis: how do CT and MRI perform in comparison to ultrasound?. PLoS Negl Trop Dis.

[33] Hosch W, Stojkovic M, Jänisch T (2008). MR imaging for diagnosing cysto-biliary fistulas in cystic echinococcosis. Eur J Radiol.

[34] Conchedda M, Caddori A, Caredda A, Capra S, Bortoletti G (2018). Degree of calcification and cyst activity in hepatic cystic echinococcosis in humans. Acta Trop.

[35] Alexiou K, Mitsos S, Fotopoulos A (2012). Complications of hydatid cysts of the liver: spiral computed tomography findings. Gastroenterol Res.

[36] Lissandrin R, Tamarozzi F, Piccoli L (2016). Factors influencing the serological response in hepatic *Echinococcus granulosus* Infection. Am J Trop Med Hyg.

[37] Stojković M, Weber TF, Junghanss T (2018). Clinical management of cystic echinococcosis: state of the art and perspectives. Curr Opin Infect Dis.

[38] Mendez Montero JV, Arrazola Garcia J, Lopez Lafuente J, Antela Lopez J, Mendez Fernandez R, Saiz Ayala A (1996). Fat-fluid level in hepatic hydatid cyst: a new sign of rupture into the biliary tree?. AJR Am J Roentgenol.

[39] Beric V, Blomley M (1997). Re: fat-fluid level in hepatic hydatid cyst: a new sign of rupture into the biliary tree?. AJR Am J Roentgenol.

[40] Collado-Aliaga J, Romero-Alegría Á, Alonso-Sardón M (2019). Complications associated with initial clinical presentation of cystic echinococcosis: a 20-year cohort analysis. Am J Trop Med Hyg.

[41] Akcan A (2010). Predisposing factors and surgical outcome of complicated liver hydatid cysts. World J Gastroenterol.

[42] Symeonidis N, Pavlidis T, Baltatzis M (2013). Complicated liver echinococcosis: 30 years of experience from an endemic area. Scand J Surg.

[43] Spârchez Z, Osian G, Onica A, Bărbântă C, Tanţău M, Pascu O (2004). Ruptured hydatid cyst of the liver with biliary obstruction: presentation of a case and review of the literature. Rom J Gastroenterol.

[44] Collado Aliaga J, Romero-Alegría Á, Alonso-Sardón M (2021). Portal hypertension as a complication of cystic echinococcosis: a 20-year cohort analysis. Am J Trop Med Hyg.

[45] Pedrosa I, Saíz A, Arrazola J, Ferreirós J, Pedrosa CS (2000). Hydatid disease: radiologic and pathologic features and complications. Radiographics.

[46] Haghighi L, Rahimi M, Behniafar H, Taghipour N, Jafari R (2021). A challenging diagnosis of two ruptured and intact pulmonary echinococcal cysts in a 54-year-old woman: a case report. Acta Parasitol.

[47] Kuzucu A, Soysal O, Ozgel M, Yologlu S (2004). Complicated hydatid cysts of the lung: clinical and therapeutic issues. Ann Thorac Surg.

[48] Wu L, Mu L, Si M, Xu J, Ciren G, Cai L (2021). Application of Multi-slice computed tomography for the preoperative diagnosis and classification of pulmonary cystic echinococcosis. Pathogens.

[49] Khuroo MS (2021). Percutaneous drainage in hepatic hydatidosis-the PAIR technique: concept, technique, and results. J Clin Exp Hepatol.

[50] Akhan O, Gumus B, Akinci D, Karcaaltincaba M, Ozmen M (2007). Diagnosis and percutaneous treatment of soft-tissue hydatid cysts. Cardiovasc Intervent Radiol.

